# Public awareness of the association between human papillomavirus and oropharyngeal cancer

**DOI:** 10.1093/eurpub/ckab081

**Published:** 2021-06-03

**Authors:** Femke Verhees, Imke Demers, Leo J Schouten, Matt Lechner, Ernst-Jan M Speel, Bernd Kremer

**Affiliations:** 1 Department of Otorhinolaryngology, Head and Neck Surgery, GROW-School for Oncology and Development Biology, Maastricht University Medical Centre, Maastricht, The Netherlands; 2 Department of Pathology, GROW-School for Oncology and Development Biology, Maastricht University Medical Centre, Maastricht, The Netherlands; 3 Department of Epidemiology, GROW-School for Oncology and Development Biology, Maastricht University Medical Centre, Maastricht, The Netherlands; 4 UCL Cancer Institute, University College London, London, UK; 5 Head and Neck Centre, University College London Hospitals NHS Trust, London, UK

## Abstract

**Background:**

Early diagnosis of human papillomavirus (HPV) associated oropharyngeal cancer (OPC) is associated with improved survival. To achieve early diagnosis, it might be beneficial to increase awareness of the link between HPV and OPC. This increase of awareness could also be an important way to increase vaccination rates. The aim of our study was to explore the current public knowledge in the Netherlands regarding the association of HPV with OPC.

**Methods:**

An online cross-sectional survey was used and sent by the company Flycatcher Internet Research to 1539 of their panel members. Data were analyzed statistically by gender, age, educational level and the participants’ use of alcohol and tobacco.

**Results:**

The response rate was 68% (1044 participants). Our data revealed that 30.6% of the participants had heard of HPV. There was a knowledge gap regarding HPV in males (*P* < 0.001), people older than 65 years (*P* < 0.001), people with low education level (*P* < 0.001) and current smokers (*P* < 0.001). Of the respondents who had heard of HPV, only 29.2% knew of the association between HPV and OPC. We also found that only 49.7% of the population knew of the existence of an HPV vaccine.

**Conclusions:**

The results of this survey indicate that the public awareness of HPV and the association of HPV with OPC is lacking. Interventions to increase awareness of HPV and its association with non-cervical cancer should be considered. This might help to increase the HPV vaccine uptake both for girls and boys and earlier diagnosis of this disease leading to improved survival.

## Introduction

Head and neck squamous cell carcinoma (HNSCC) has been the seventh most common cancer worldwide in 2018, accounting for 3% of all cancers.^1^ The majority of HNSCC cases are tobacco and alcohol associated, but research in the past decades has highlighted the increasing importance of human papillomavirus (HPV) infection as a risk factor for developing HNSCC, especially for oropharyngeal carcinomas (OPC).^2^ While the incidence of tobacco related disease has declined in the past two decades, there is an increase in HPV associated OPC.^2^^,^^3^ The HPV associated oropharyngeal tumors have different properties than the HPV negative HNSCC; patients are younger, more often male and non-smokers and non-drinkers. In addition, HPV associated OPC is more often seen in population with a higher socio-economic class.^4^ Individuals with frequent oral sex encounters, a greater number of different sexual partners, and earlier sexual experiences seem to be at a higher risk for HPV associated OPC development.^5–7^ Earlier diagnosis of HPV associated OPC is associated with improved survival.^8^ To achieve early diagnosis, it might be beneficial to increase awareness of the link between HPV and OPC.

Recent data in the United States suggests that the incidence of HPV related OPC exceeds the incidence of HPV related cervical cancer in high income countries, although some reservations must be made because of regional differences.^9^^,^^10^ The HPV vaccine not only protects against the development of cervical cancer, but also against oropharyngeal cancer.^11^ In the Netherlands, since 2009 girls aged 13 years have been offered an HPV vaccination to prevent cervical cancer development from the National Vaccination Program.^12^ The vaccine has been included in the vaccination program for boys since the beginning of 2021. Children will also be vaccinated at a younger age from 2021, namely from the age of 9. To maximize the potential benefits of HPV vaccination, it is necessary to get the vaccination coverage as high as possible. The national vaccination coverage for HPV for girls was 53% in the Netherlands in 2019.^13^ Because the parents decide on the vaccination, it is important that they are aware of the association between HPV and not only cervical cancer, but also OPC.

Since vaccination against HPV became available, awareness of HPV has dramatically increased.^14^ A study by Williams et al.^15^ under the general public in the United States showed that most respondents were aware that HPV is a causative agent of cervical cancer. However, the majority were not aware of the association between HPV and oropharyngeal cancer. Data from a recent study regarding the public awareness of HPV associated oropharyngeal cancer in men and women in the United Kingdom showed that 37% of the respondents had ever heard of HPV and of these 38.7% recognized HPV as a risk factor for OPC.^16^

The aim of our study was to explore the current public knowledge in the Netherlands regarding the association of HPV with oropharyngeal cancer. Our findings will help us to determine if there is need to increase public education on HPV and oropharyngeal cancer. By increasing education and uptake of the HPV vaccine, we hope to combat the development of HPV associated oropharyngeal cancers and other HPV associated tumors.

## Methods

### Survey design and administration

The medical ethics review committee of Maastricht University Medical Centre approval was obtained on the basis that data collection was anonymized and no vulnerable participants were involved.

A short questionnaire was already developed by Lechner et al.,^16^,^17^ which was kindly provided to us and which we have adapted to our situation. The questionnaire of nine items (see Supplementary data) assessed the knowledge of HPV, of OPC risk factors and symptoms, of the association between HPV and OPC, the knowledge of the HPV vaccine and the participants’ use of alcohol and tobacco. Tobacco use was divided into current user, former smoker, and non-smoker (never smoked), and alcohol consumption was classified in 1–7 drinks per week, 8–14 drinks per week, 15–21 drinks per week, more than 21 drinks per week or no drinks. Demographic characteristics of the participants were provided to us by the company Flycatcher Internet Research, as they sent the online questionnaire to their panel members. These characteristics included gender, age, education level and living in which province. Education level was measured as low, middle and high. Low was defined as having no certificate or having a certificate of pre-vocational secondary education or secondary vocational education. Middle was defined as having a certificate of intermediate vocational education, or senior general secondary education or pre-university education or having a first year’s degree in higher professional education or in university education. High was defined as having a certificate of higher professional education or of university education or having a doctoral or post-doctoral degree.

The company Flycatcher Internet Research sent the online questionnaire to the research group selected from a sample from their panel consisting of people older than 18 years who have registered voluntarily. The sample was stratified by gender, age, educational level and province. This guarantees that the people in the sample were a representative reflection of the Dutch population aged 18 and older. The selected panelists received an e-mail describing the study, and interested respondents were directed to a website where the survey could be completed. The intended response rate was 1000 participants. Respondents were encouraged to completely fill out the whole survey. Incompletely filled surveys were excluded in the analysis.

### Statistical analysis

Statistical analyses were performed using SPSS statistical software for Windows, version 25 (IBM). Descriptive analyses with calculated measures of central tendency and variation were computed, along with frequency tables for categorical variables. Whether distributions of categories are different was tested using Chi-square test. The significance level was set at *P* = 0.05.

## Results

### Participant characteristics

The online questionnaire was sent to 1539 panel members, of whom 1044 completed the questionnaire (response rate 68%). In 16 other questionnaires, one or more questions were skipped and therefore excluded.

This population reflected the Dutch population in terms of gender, age, education level and province. The characteristics of the participants are shown in table 1.

**Table 1:** Characteristics of the participants (*N* = 1044)

Characteristics	*N*	%
Sex		
Male	517	49.0
Female	527	51.0
Age		
18–29 years	173	17.0
30–65 years	590	56.0
>65 years	281	27.0
Educational level		
Low	293	28.0
Middle	463	44.0
High	288	28.0
Smoking		
Non-smoker	491	47.0
Former smoker	426	41.0
Current smoker	127	12.0
Alcohol = drinks per week		
No alcohol use	382	37.0
1–7 drinks	504	48.0
8–14 drinks	110	11.0
15–21 drinks	34	3.0
>21 drinks	14	1.0

### Knowledge of HPV

Of the 1044 respondents, 30.6% had ever heard of HPV (table 2). Two times more women were aware of HPV than men (41.6% vs. 19.3% *P* < 0.001). Participants aged 18–29 years had most often heard of HPV (44.5%) and participants over 65 years the least (10.7%) (*P* < 0.001). Participants with a low educational level had heard of HPV less often than participants with a high education level (12.3% vs. 46.9%) (*P* < 0.001). Participants who did not smoke more frequently had heard about HPV than those who smoked or had smoked (38.5% vs. 18.9% and 24.9% *P* < 0.001). Of the respondents who already had heard of HPV, 79.9% knew that HPV is transmitted during sex, 72.7% that HPV is transmitted during oral sex, 78.4% that HPV is not rare and only 64.6% knew that HPV does not cause HIV (table 3).

**Table 2:** Knowledge about HPV and oropharyngeal cancer in the Dutch population (*N* = 1044)

Characteristics	Yes. I had heard of HPV before today			Yes. I'm aware of an HPV vaccine			Yes. I'm aware of an HPV vaccine AND I knew of HPV			Yes. I knew of the link between HPV and OPC			Yes. I knew of the link between HPV and OPC AND I knew of HPV		
	*N*	%	*P*-value	*N*	%	*P*-value	*N*	%	*P*-value	*N*	%	*P*-value	*N*	%	*P*-value
	319	30.6		519	49.7		262	82.1^a^		115	11.0		93	29.2	
Sex															
Male	100	19.3	<0.001	202	39.1	<0.001	75	75.0	0.013	47	9.1	0.049	34	34.0	0.20
Female	219	41.6		317	60.2		187	85.4		68	12.9		59	26.9	
Age															
18–29 years	77	44.5	<0.001	101	58.4	<0.001	62	80.5	0.008	26	15.0	0.008	22	28.6	0.87
30–65 years	212	35.9		313	53.1		179	84.4		71	12.0		61	28.8	
> 65 years	30	10.7		105	37.4		21	70.0		18	6.4		10	33.3	
Educational level															
Low	36	12.3	<0.001	118	40.3	<0.001	29	80.6	0.046	19	6.5	0.002	10	27.8	0.92
Middle	148	32.0		219	47.3		115	77.7		51	11.0		42	28.4	
High	135	46.9		182	63.2		118	87.4		45	15.6		41	30.4	
Smoking															
Current smoker	24	18.9	<0.001	47	37.0	0.004	14	58.3	0.011	5	3.9	0.001	4	16.7	0.36
Former smoker	106	24.9		202	47.4		86	81.8		39	9.2		31	29.2	
Non-smoker	189	38.5		270	55.0		162	85.7		71	14.5		58	30.7	
Alcohol = drinks per week															
1–7 drinks	171	33.9	0.076	263	52.2	0.041	140	81.9	0.24	60	11.9	0.303	51	29.8	0.96
8–14 drinks	22	20.0		50	45.5		16	72.7		8	7.3		7	31.8	
15–21 drinks	7	20.6		14	41.2		6	85.7		2	5.9		2	28.6	
>21 drinks	1	7.1		2	14.3		0	0.0		0	0.0		0	0.0	
No alcohol use	118	30.9		190	49.7		100	84.7		45	11.8		33	28.0	

a Percentage of participants who were aware of an HPV vaccine and did NOT heard of HPV before today = 34.5%.

Note: HPV, human papillomavirus; OPC, oropharyngeal cancer.

**Table 3:** Knowledge about HPV when already heard of HPV (*N* = 319)

	Yes		No		Not sure	
	*N*	%	*N*	%	*N*	%
Is HPV rare?	20	6.3	250	78.4	49	15.4
Is HPV transmitted during sex?	255	79.9	29	9.1	35	11.0
Is HPV transmitted during oral sex?	232	72.7	30	9.4	57	17.9
Can HPV cause HIV (Aids)?	22	6.9	206	64.6	91	28.5

Note: HPV, human papillomavirus; HIV, Human immunodeficiency virus.

### Knowledge about HPV vaccine

Despite knowledge of HPV in 30.6% (*N* = 319) of all participants (mentioned above), we found that 49.7% (*N* = 519) of all participants knew that there is an HPV vaccine available. This is remarkable, because this means that a part of the participants who had no knowledge of HPV knew that there is a vaccine (table 2). Participants older than 65 years were less aware of HPV vaccination (70%, *P* = 0.008), but there was less spread in the knowledge of the HPV vaccine between the different education levels. Current smokers and participants drinking more than 21 alcoholic drinks per week were also less aware of the existence of an HPV vaccine (58.3% and 0%, respectively), although the latter group was small (14 persons).

### Knowledge about oropharyngeal cancer

In the overall population, 11% knew of the association between HPV and OPC. Interestingly, of the respondents who had heard of HPV, only 29.2% recognized HPV as risk factor of OPC (table 2). In comparison to the knowledge of the existence of HPV, men were now more aware of this link than women (34.0% vs. 26.9% *P* = 0.20), but the knowledge of the link was more equal across the different age categories and education levels. Because parents decide whether or not their children will undergo HPV vaccination, we also looked specifically at the participants aged 30–45 years for the knowledge about HPV and OPC. This knowledge was not different from the participants aged 45–65 years (data not shown). Current smokers and participants drinking more than 21 alcoholic drinks per week were again less aware of the link between HPV and OPC (16.7% and 0%, respectively).

Participants were confronted with 11 factors and asked whether these were risk factors for OPC or not. Only 26.9% of the participants correctly identified HPV as a risk factor for OPC (table 4), which is higher than the initial 11.0% (mentioned above). Awareness of other well-established risk factors was much higher: for example, smoking (97.3%) and chewing tobacco (74.5%). Excessive alcohol consumption, poor oral hygiene and chewing of betel leaf, catchu and areca nuts were less recognized (60%, 38.1% and 30.4%, respectively).

**Table 4:** Knowledge of reported risk factor for oropharyngeal cancer in the general Dutch population (*N* = 1044)

Risk factor	Yes		No		Not sure	
	*N*	%	*N*	%	*N*	%
Excessive alcohol consumption	626	60.0	139	13.3	279	26.7
Smoking	1016	97.3	10	1.0	18	1.7
Chewing of tobacco	778	74.5	48	4.6	218	20.9
Chewing of betel leaf. catchu and areca nuts	317	30.4	87	8.3	640	61.3
Marijuana use	547	52.4	109	10.4	388	37.2
Poor oral hygiene	398	38.1	274	26.2	372	25.6
Herpes simplex virus infection	277	26.5	139	13.3	628	60.2
Human papilloma virus infection	281	26.9	112	10.7	651	62.4
Family history of cancer	646	61.9	136	13.0	262	25.1
Low fruit and vegetable consumption	253	24.2	338	32.4	453	43.4
Sun exposure	167	16.0	454	43.5	423	40.5

Before this question, the participants were asked with an open question what they think could affect a person’s chance of throat cancer. Notable factors mentioned include poor air quality (94 times), harmful chemicals (84 times), hot drinking (42 times) and spicy food (17 times).

## Discussion

Over the past three decades, there has been a clear decrease in the prevalence of tobacco use and an associated decline in tobacco related head and neck cancers in many industrialized countries. The incidence of HPV positive OPC, however, is increasing worldwide, predominantly among men.^2^^,^^3^ Recent data in the United States suggests that the incidence of HPV related OPC exceeds the incidence of HPV related cervical cancer in high income countries, although some reservations must be made because of regional differences.^9^ The HPV vaccine not only protects against cervical cancer, but also against oropharyngeal cancer.^11^ Several studies suggested that the public is relatively well informed about HPV as a sexually transmitted disease and of the relationship between HPV and cervical cancer.^14^ In contrast, there seems to be a lack of knowledge about the association of HPV and OPC.^15^^,^^16^

The present study focused on the awareness of the Dutch population concerning the association between HPV and OPC. Our data revealed that 30.6% of the population had heard of HPV and that this knowledge was less in males, people older than 65 years, low education level and current smokers. Of the respondents who had heard of HPV, only 29.2% knew of the association between HPV and OPC. This frequency is slightly lower in comparison with earlier studies, for example the study of Williams et al.^15^, in which 36% of the respondents reported to know that HPV is a causative factor for OPC. An explanation could be the fact that more than 75% of the participants in the study of Williams were aged between 18 and 35, while in our study only 17% of the respondents were aged 18–29 years and 56% aged 30–65 years. In the study of Lechner et al.^16^ however, 38.7% of the respondents knew of the association between HPV and OPC and the age range of the participants was comparable with that in our study. The participants of our study who were aware of HPV were in general well aware of the prevalence and the (oral) sexually transmission of HPV.

We also found that 49.7% of the population knew of the existence of an HPV vaccine, this percentage was remarkable because it is higher than the percentage of the population knowing of the virus itself. So, 34.5% of the respondents who had never heard of HPV were aware of the presence of an HPV vaccine. One explanation for this difference could be that the addition of ‘vaccine’ to ‘HPV’ increases the knowledge because it creates an association, which people have less with the word ‘HPV’ alone. Another explanation could be that people don’t know what the HPV vaccine is for. In addition, it was striking that if we asked in an open question whether the participants knew about the link between HPV and OPC, only 11% answered positively, whereas when we presented the respondents a list of causative factors for OPC, 26.9% indicated that there was an association between HPV and OPC. We think this is because of the respectively closed versus open way of asking the question.

The greater awareness among women about HPV, the HPV vaccine and the link of HPV with OPC, suggest that this knowledge is primarily due to awareness of the role of HPV in uterine cervical cancer. Since the incidence of HPV related OPC is 3–6 times higher in men than in women and the HPV related OPC exceeds the incidence of HPV related cervical cancer in the higher income countries,^3^^,^^18^ greater awareness of the role of HPV infection in OPC is necessary to improve vaccine uptake, in women but especially also in men.

The knowledge about the association between HPV and OPC was highest in the group with a higher education level and among non-smokers and non-drinkers. This is beneficial because this group has the highest risk of getting HPV associated OPC. However, in general, the knowledge is still substantially low so that more awareness is needed. In addition, greater awareness of the disease may prompt patients harboring symptoms of HPV positive cancers to go in time to the physician. Subsequently, the physician must be sufficiently aware of symptoms and risk factors of OPC. A recent study by Lechner et al.^17^ reported that the level of awareness of HPV and OPC among general practitioners was high; however, the characteristics of HPV associated OPC were less well recognized, indicating the need for further education. Therefore, studying the awareness of HPV and OPC, other risk factors and symptoms among the general practitioners in the Netherlands should also be considered.

There are some limitations of this study that should be considered when interpreting its results. All Internet-based surveys incur the potential for bias by excluding participants who lack Internet connections.^19^ Moreover, in this particular study there is also the potential for bias because of the selection of people who want to participate in a panel. Internet surveys are also vulnerable for bias due to nonresponse. As a consequence, the participants may differ significantly from the general population.^20^ However, the results of this survey are largely consistent with previously published data on HPV awareness.^15^^,^^16^^,^^21^

This survey was conducted during the COVID pandemic, which may result in an increased interest in virus vaccines and could therefore have influenced the response rate.

In conclusion, the results of this survey indicate that the public awareness of HPV and the association of HPV with oropharyngeal cancer is lacking. Interventions to increase awareness of HPV and its association with non-cervical cancer should be considered. This might help to increase the HPV vaccination uptake and earlier diagnosis of this disease leading to improved survival.

## Supplementary data

Supplementary data are available at *EURPUB* online. 

## Conflict of interest

E.-J.S. reports grants from Pfizer and Novartis and honoraria from BMS. The funders had no role in this particular study design; in the collection, analyses, or interpretation of the data; in the writing of the manuscript or in the decision to publish the results. The other authors declare no conflict of interest.

**Key points**

- This study is the first to examine awareness on human papillomavirus (HPV)-associated oropharyngeal cancer (OPC) among the Dutch population.
- 30.6% of the participants had heard of human papillomavirus (HPV) and only 29.2% of these participants knew about the association between HPV and oropharyngeal cancer.
- The results of this survey indicate that the public awareness of HPV and the association with oropharyngeal cancer is lacking.
- Interventions to increase this knowledge might help to increase the HPV vaccination uptake and earlier diagnosis of this disease leading to improved survival.

## Supplementary Material

ckab081_Supplementary_Data
