# Supplementary material for: Public awareness of the association between human papillomavirus and oropharyngeal cancer
Source: Eur J Public Health. 2021 Jun 3;31(5):1021–5. doi: 10.1093/eurpub/ckab081 (PMC8565482; doi:10.1093/eurpub/ckab081)
Supplement: ckab081_Supplementary_Data [file ckab081_supplementary_data.pdf]

## Questionnaire about throat cancer

**1. What things do you think affect a person's chance of throat cancer? If you cannot think of any, please "don't know" in the box below.**

| |
|---|
| |

**2. Which of the following are common factors for an increased risk of getting throat cancer?**

| | Yes | No | Not sure |
|---|---|---|---|
| Excessive alcohol consumption | o | o | o |
| Smoking | o | o | o |
| Chewing of tobacco | o | o | o |
| Chewing of Betel leaf/ Catchu and areca nuts | o | o | o |
| Marijuana use | o | o | o |
| Poor oral hygiene | o | o | o |
| Herpes simplex virus infection | o | o | o |
| Human papillomavirus infection | o | o | o |
| Family history of cancer | o | o | o |
| Fruit and vegetable consumption | o | o | o |
| Sun exposure | o | o | o |

**3. There are many warning signs and symptoms of throat cancer. Please name as many as you can. If you cannot think of any, please type "don't know" in the box below.**

| |
|---|
| |

**4. Before today had you ever heard of HPV (human papillomavirus)?**

- o Yes
- o No
- o Not sure

HPV is the virus that causes cervical cancer.

**5. Please read the following statements and say whether you think each one is true or false**

| | True | False | Not sure |
|---|---|---|---|
| HPV is very rare | o | o | o |
| HPV can be passed on during sex | o | o | o |
| HPV can be passed on during oral sex | o | o | o |
| HPV can cause HIV/AIDS | o | o | o |
| There is a vaccine against the virus HPV | o | o | o |

**6. Were you aware that the virus HPV (human papillomavirus) is a risk factor for throat cancer?**

- o Yes
- o No

Finally, a number of background questions follow below.

**7. Do you smoke?**
- Yes, I'm a current smoker
- Yes, I have smoked in the past
- No

**8. How many cigarettes do you smoke per day?**
- Less than 10 per day
- 10 - 19 per day
- 20 - 35 per day
- 35 of more per day

**9. How many units of alcohol do you consume in the average week?**
- 1-7
- 8-14
- 15-21
- More than 21
- I never drink alcohol
